# Supplementary material for: Association between oral microbiome and seven types of cancers in East Asian population: a two-sample Mendelian randomization analysis
Source: Front Mol Biosci. 2023 Nov 21;10:1327893. doi: 10.3389/fmolb.2023.1327893 (PMC10702768; doi:10.3389/fmolb.2023.1327893)
Supplement: Supplementary file 1 [file DataSheet1.ZIP › Supplementary Materials/MR plots of saliva/saliva═╝/Gastric cancer/pheno.3565_to_gastric cancer_leave_one_out.pdf]

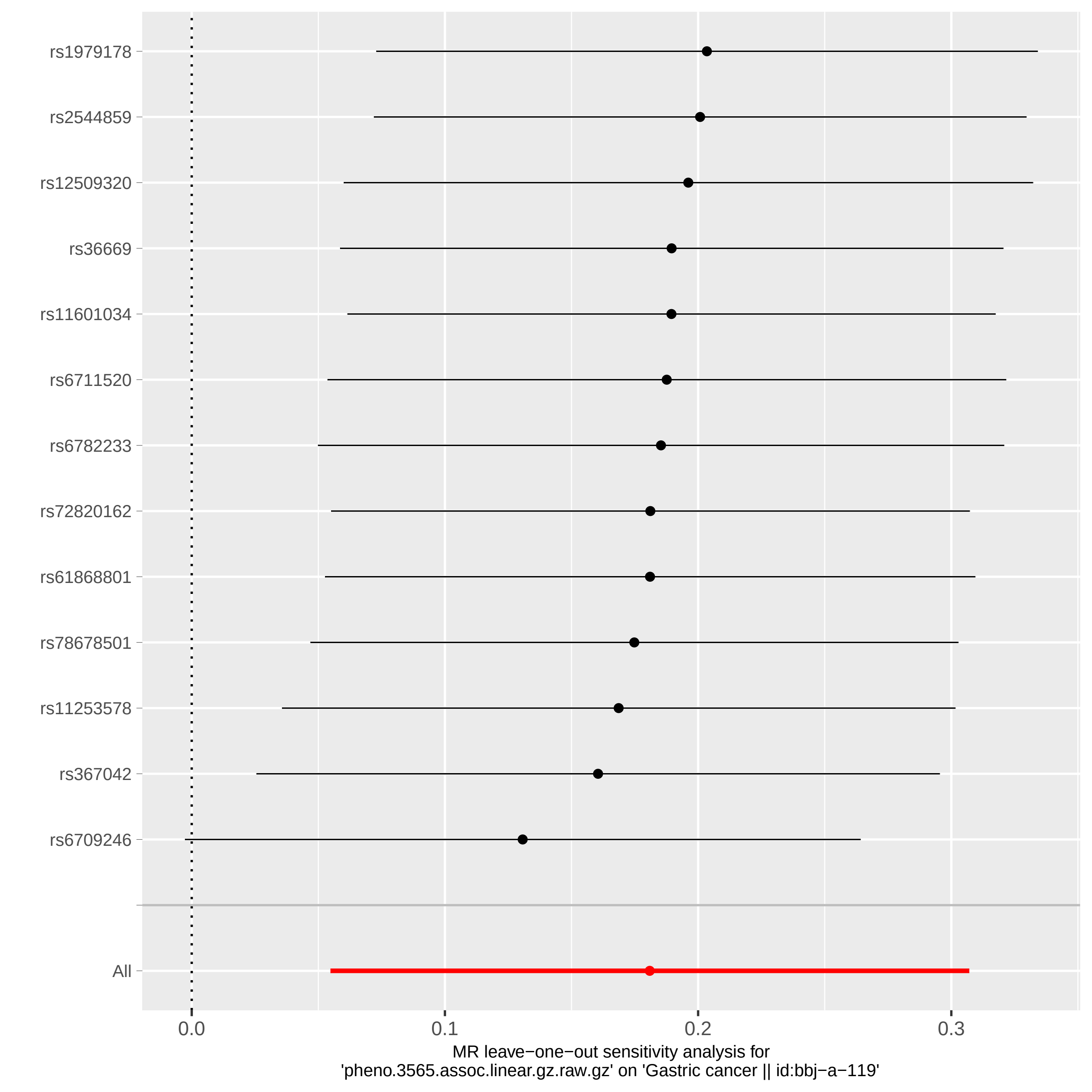
rs1979178
rs2544859
rs12509320
rs36669
rs11601034
rs6711520
rs6782233
rs72820162
rs61868801
rs78678501
rs11253578
rs367042
rs6709246
All
0.0
0.1
0.2
0.3
MR leave−one−out sensitivity analysis for
'pheno.3565.assoc.linear.gz.raw.gz' on 'Gastric cancer || id:bbj−a−119'
